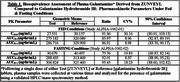# Bioequivalence of ZUNVEYL a Galantamine Prodrug to Galantamine Hydrobromide Immediate‐Release Demonstrated Under Fed and Fasting Conditions

**DOI:** 10.1002/alz70859_107030

**Published:** 2025-12-25

**Authors:** Denis G Kay, Kurt P Grady, Andrew J Wahlert

**Affiliations:** ^1^ Alpha Cognition Inc, Vancouver, BC Canada

## Abstract

**Background:**

ZUNVEYL (benzgalantamine, galantamine benzoate gluconate), is a pharmacologically inactive prodrug of galantamine. ZUNVEYL was FDA approved in 2024 for BID dosing for the treatment of mild to moderate Alzheimer’s dementia, via the 505(b)(2) regulatory pathway, relying on FDA’s previous finding of safety and efficacy for the listed drugs (LDs) Razadyne® (galantamine hydrobromide tablets), and Razadyne® ER (galantamine hydrobromide extended‐release capsules). When dosed as a delayed‐release (DR) tablet, ZUNVEZYL bypasses the stomach and, is absorbed in the small intestine potentially reducing the gastrointestinal side effects common for acetylcholinesterase inhibitors. Consequently, ZUNVEYL may offer advantages over the other acetylcholinesterase inhibitors by reducing gastrointestinal adverse effects which limit compliance. Here we describe the outcomes of two studies designed to assess the relative bioavailability of ZUNVEYL DR tablets to galantamine hydrobromide immediate release (IR) tablets under fed and fasting conditions.

**Methods:**

The studies were open‐label, balanced, randomized, single‐dose, two‐treatment, two‐period, two‐way crossover designs to evaluate the relative bioavailability of ZUNVEYL DR 5 mg tablet compared to galantamine hydrobromide 4 mg IR tablet, in healthy adult subjects under fed (Study ALPHA‐1062‐01; N=34) and fasting (Study ALPHA‐1062‐02; N=34) conditions. Study protocols underwent ethics review and were conducted to GCP standards.

**Results:**

Table 1 documents plasma pharmacokinetic parameters calculated from the analysis of galantamine (from ZUNVEYL) and galantamine hydrobromide IR. ZUNVEYL (prodrug) was inconsistently detected in plasma and represented approximately 1% of total circulating drug. The low percentage of circulating prodrug provides a greater margin of safety.

**Conclusions:**

ZUNVEYL was well‐tolerated with no serious adverse events noted. Bioequivalence assessment demonstrated that ZUNVEYL was bioequivalent to galantamine hydrobromide IR for AUC0‐t and AUC0‐∞, under fed and fasting conditions, and for Cmax under fed conditions. These results combined with those of a steady state study [abstract # 107147], established a scientific bridge to the LDs and the approval of ZUNVEYL, relying on FDA’s previous finding of safety and efficacy of the LDs.